# Molecular Epidemiology of Tuberculosis in Finland, 2008-2011

**DOI:** 10.1371/journal.pone.0085027

**Published:** 2013-12-26

**Authors:** Pieter Willem Smit, Marjo Haanperä, Pirre Rantala, David Couvin, Outi Lyytikäinen, Nalin Rastogi, Petri Ruutu, Hanna Soini

**Affiliations:** 1 European Public Health Microbiology Training Programme, (EUPHEM), European Centre for Disease Prevention and Control (ECDC), Stockholm, Sweden; 2 Department of Infectious Disease Surveillance and Control, National Institute for Health and Welfare, Helsinki, Finland; 3 WHO Supranational TB Reference Laboratory, Tuberculosis & Mycobacteria Unit, Institut Pasteur de la Guadeloupe, Abymes, France; St. Petersburg Pasteur Institute, Russian Federation

## Abstract

In industrialized countries the majority of tuberculosis (TB) cases are linked to immigration. In Finland, most cases are still Finnish born but the number of foreign born cases is steadily increasing. In this 4-year population based study, the TB situation in Finland was characterized by a genotypic analysis of *Mycobacterium tuberculosis* isolates. A total of 1048 *M. tuberculosis* isolates (representing 99.4% of all culture positive cases) were analyzed by spoligotyping and MIRU. Spoligotype lineages belonging to the Euro-American family were predominant among the Finnish isolates, particularly T (n=346, 33.0%) and Haarlem (n=237, 22.6%) strains. The lineage signature was unknown for 130 (12.4%) isolates. Out of the 17 multi-drug resistant TB strains, 10 (58.8%) belonged to the Beijing lineage. In total, 23 new SIT designations were given and 51 orphan strains were found, of which 58 patterns were unique to Finland. Phylogeographical TB mapping as compared to neighboring countries showed that the population structure in Finland most closely resembled that observed in Sweden. By combining spoligotyping and MIRU results, 98 clusters comprising 355 isolates (33.9%) were found. Only 10 clusters contained both Finnish and foreign born cases. In conclusion, a large proportion of the *M. tuberculosis* isolates were from Finnish born elderly patients. Moreover, many previously unidentified spoligotype profiles and isolates belonging to unknown lineages were encountered.

## Introduction

Finland had a very severe tuberculosis (TB) epidemic in the beginning of the 20^th^ century, with respiratory TB mortality rates of 250-300 per 100,000 [1]. After the Second World War, TB mortality rates started to decline reaching 51 per 100,000 in 1951. This rapid decrease was related to an improved standard of living, effective TB control program and availability of TB medication [1]. In 2001, Finland became a low incidence country when TB incidence fell below 10 per 100,000, and in 2012 the TB incidence was 5/100,000. 

However, the epidemiological situation in Finland is different from neighboring low incidence countries. In Sweden, Norway and Denmark, a transition took place from the native population to foreigners, that currently account up to 80% of the new TB cases [2–4]. In Finland, most cases are still Finnish born but the number of foreign born cases is steadily increasing over time. In our neighboring countries in the East and South, the TB incidence is high and predominantly associated with *Mycobacterium tuberculosis* isolates belonging to the Beijing genotype family [5]. Multidrug resistant (MDR) TB is a significant problem in Russia and Estonia, while in Finland this remains a rare occurrence [6–8]. Puustinen et al. showed that *M. tuberculosis* isolates belonging to the Beijing lineage were infrequently encountered in Finland [9]. However, MDR-TB cases will most likely increase in Finland as well, because of the rapid spread of the Beijing lineage across Europe and increasing immigration to Finland. 

To better understand the current epidemiology of TB in a country with a history of high TB incidence, the aim of the study was to evaluate the current TB situation in Finland by genotypic characterization of *M. tuberculosis* complex isolates. Our first objective was to use the typing data to provide a general overview of the genotypes retrieved from TB cases in Finland, and compare the situation in Finland to our neighboring countries. 

## Materials and Methods

Since 1995, the reporting of approximately 70 infectious diseases, including TB, to the National Infectious Disease Registry has been mandatory for physicians and clinical microbiology laboratories in Finland [8]. In addition, all *M. tuberculosis* complex isolates are sent to the Mycobacterial Reference Laboratory at the National Institute for Health and Welfare (THL) for species confirmation, genotyping, drug susceptibility testing, and strain collection.

### Isolates

Between 2008 and 2011, 1397 patients were notified with TB, and cultures with *M. tuberculosis* complex were obtained from 1054 patients. The data in this study were analyzed within the epidemiological research purposes authorized by the Finnish Communicable Diseases Act (clause 4 in section 40a, Finlex, Valtion säädöstietopankki. Available at: http://www.finlex.fi/fi/laki/alkup/2003/20030935 (accessed 23.08.2013). Therefore, ethical approval was deemed unnecessary. 

From the culture positive patients with a valid personal identification code (Finnish social security number), 1048 (99.4%) isolates were successfully genotyped at the reference laboratory, and included in this study. The isolates were cultured on Löwenstein – Jensen medium for further analysis. All isolates were characterized by spoligotyping, as described previously [9]. The spoligotype patterns were uploaded and assignment of the Spoligo International Type (SIT) designations was done using the international SITVIT2 database of the Institute Pasteur of Guadeloupe, which is an updated version of the SITVITWEB database [10]. When a spoligotype was not found in the SITVIT2 database, it was classified as a unique spoligotype. The term “orphan spoligotype” refers to an isolate having a spoligotype that was present only once in our dataset. When multiple isolates (i.e. 2 or more) were obtained with a unique spoligotype pattern, a new SIT number was assigned by Institute Pasteur. For the unique patterns, lineage designations were given manually by expert-based interpretations using revised SpolDB4 rules. A Finnish designation “F-type” was assigned to the Finnish unique orphan spoligotypes. Fifteen loci mycobacterial interspersed repetitive units -variable number of tandem repeats (MIRU VNTR (MIRU)) was performed at Genoscreen (Lille, France) on all isolates. 

Genotypic lineages (i.e. clades or families) were obtained from the SITVIT2 database (accession date: May 16^th^ 2013), designated in the database as: the Beijing clade, the Central Asian (CAS) clade and 2 sublineages, the East African-Indian (EAI) clade and 9 sublineages, the Haarlem (H) clade and 3 sublineages, the Latin American-Mediterranean (LAM) clade and 12 sublineages, the ancestral “Manu” family and 3 sublineages, the S clade, the IS*6110*-low-banding X clade and 3 sublineages, an ill-defined T clade with 5 sublineages, as well as the recently described Ural lineage (with 2 sublineages Ural-1 and Ural-2).

### Statistical analysis

Data regarding patients, clinical presentation, and *M. tuberculosis* complex isolates were obtained from the laboratory database and from the National Infectious Diseases Registry, and analyzed in STATA 12 (Statacorp LP, USA). When multiple samples were collected from the same patient, the first isolate with a genotype result was used. Spoligotypes in binary format and MIRU results were entered into BioNumerics software version 6.6 (Applied Maths Inc., Sint-Martens-Latem, Belgium) for cluster analysis. Minimum spanning tree (MST) was constructed by permutation resampling (200x) with highest resampling proportion. Fischer exact and Pearson Chi_2_ test were performed to assess the similarity between lineages. 

## Results

Of the 1048 patients, 733 (69.9%) were born in Finland, 264 (25.2%) were foreign born and for 51 (4.9%) patients the place of birth was unknown. 481 patients were ≥65 years old (45.9%) and the median age of the patients was 61 years (range: 0-103), and 601 (57.3%) patients were male. 

### Bacterial isolates

Of the 1048 *M. tuberculosis* complex isolates, 1044 (99.6%) were *M. tuberculosis*, three were *Mycobacterium africanum*, and one was *Mycobacterium bovis*. With MIRU typing, 722 distinct profiles were obtained of which 149 (20.6%, 475 isolates) were recognized and 573 (79.4%) were not recognized in the MIRU-VNTR plus website [11].From the 273 distinct spoligotypes that were identified, 215 (78.7%) were recognized in the SITVIT2 database while 58 (21.2%) were not recognized (unique). Out of the 58 unique spoligotypes, 23 new SITs (41 isolates) were created using revised SpolDB4 rules. Of the new SITs and unique orphanspoligotype patterns, 68 isolates were only detected in Finland (F-type), 67.6% (46/68) of these were obtained from Finnish born patients. 51 strains remained orphan. An overview of these strains can be found in Table S1 and Figure S1. 

### *M. tuberculosis* genotypic lineages

**Figure 1:**
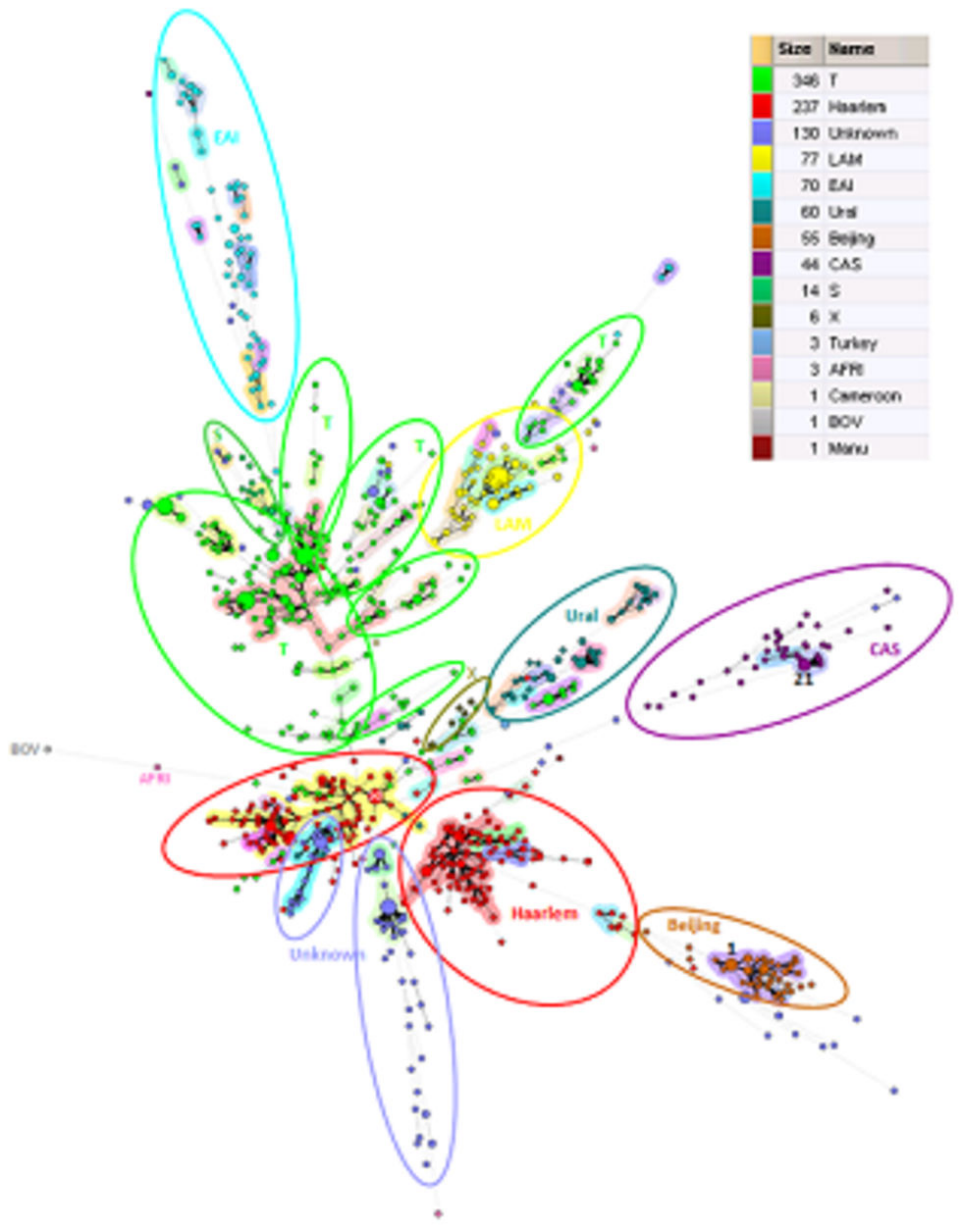
Cluster analysis of all isolates found in this study. Colors of nodes depict the lineage of the strain.

**Figure 2:**
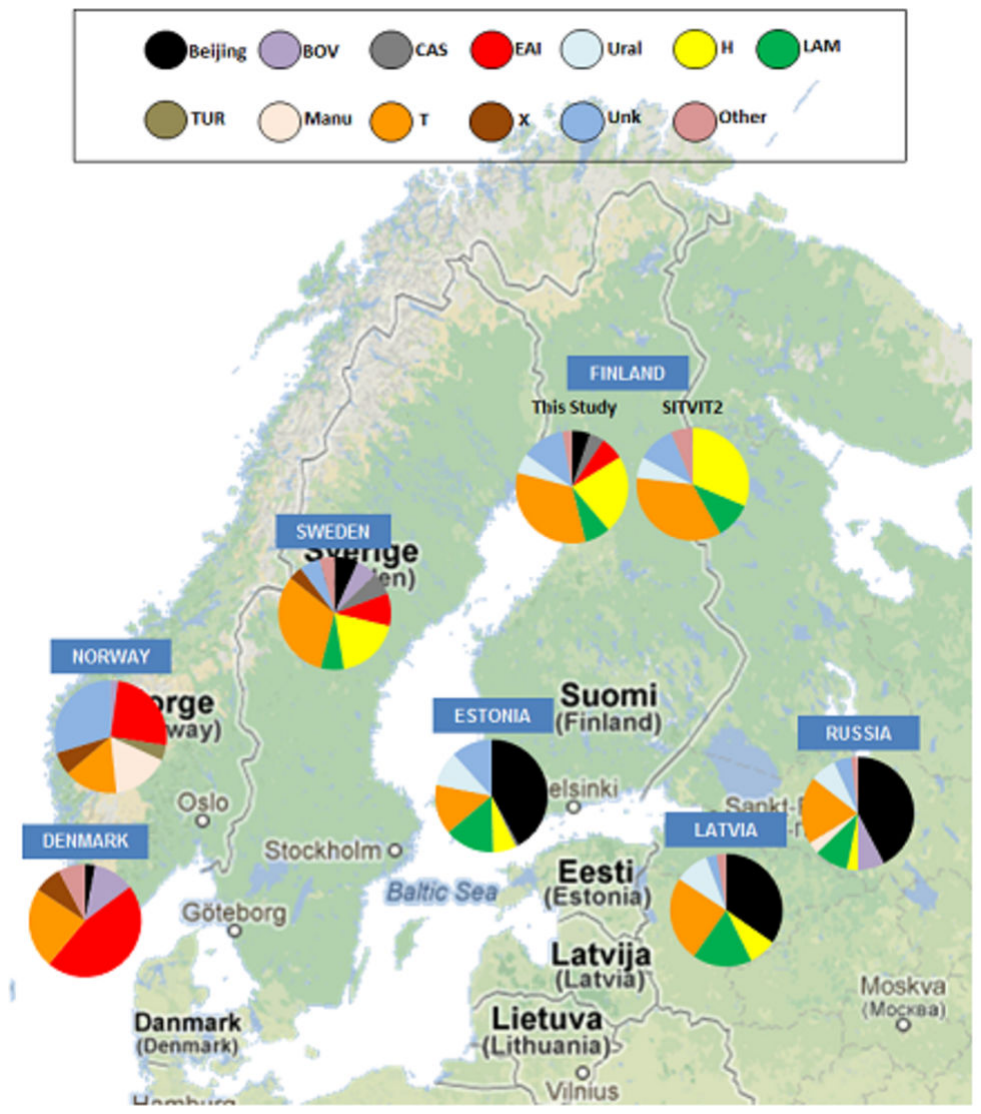
Phylogeographical distribution of *M. tuberculosis* lineages in neighboring countries. Colors of nodes depict the lineage of the strain. Note that the proportion of Ural lineage (notably Ural-1) is not negligible in the following countries: Russia, Latvia, Estonia, and Finland [11].

The most common TB lineages found from the Finnish isolates were T (n=346, 33.0%) and Haarlem (n=237, 22.6%) (Table 1 and Figure 1). For 130 isolates (12.4%) the lineage could not be defined (i.e. was unknown). Out of the 130 isolates, 103 (79.2%) were obtained from Finnish born patients. Based on phylogeographical mapping, the distribution of lineages closely resembles the distribution seen in Sweden (Figure 2). The main difference was for the Ural lineage, which according to the SITVIT2 database, was not regularly found in Sweden. Both Ural-1 and Ural-2 (42 and 18 isolates respectively) were predominantly found among Finnish born cases (50/60, 83.3%). Additionally, isolates not belonging to any known lineage (i.e. undefined clade) were found more commonly among Finnish born (n=103, 79.2%) than foreign born patients (n=25, 19.2%) (p: <0.05).

**Table 1:** An overview of the lineages found in Finland, 2008-2011.

Lineage	**Total**	**Finnish-born**	**Foreign-born**	**Unknown origin**	**Median age**	**Pulmonary**	**Extra-pulmonary**	**Pan-susceptible**	**Drug resistant**	**MDR**
**AFRI**	3	0	2	1	33	1	2	3	0	0
**Beijing**	55	11	40	4	34	41	14	31	14	10
**BOV**	1	0	1	0	25	0	1	0	1	0
**Cameroon**	1	0	1	0	42	1	0	1	0	0
**CAS**	44	6	33	5	27	22	22	33	8	3
**EAI**	70	5	53	12	31	34	36	53	17	0
**Haarlem**	237	214	20	3	73	143	94	217	19	0
**LAM**	77	65	10	2	55	57	20	73	3	1
**Manu**	1	0	1	0	24	0	1	1	0	0
**S**	14	6	5	3	50	7	7	12	1	1
**T**	346	268	63	15	66	201	145	325	19	1
**Turkey**	3	3	0	0	64	0	3	3	0	0
**Unknown**	130	103	25	2	64	75	55	115	14	1
**Ural**	60	50	6	4	71	40	20	56	4	0
**X**	6	2	4	0	66	3	3	6	0	0
	1048	733	264	51		625	423	929	100	17

A minimum spanning tree using spoligotyping and MIRU as categorical data shows the phylogenetic similarity of the *M. tuberculosis* strains. The 68 isolates with a spoligotype only found in Finland (F-type) were mostly unlinked and seemed phylogenetically remote to SIT strains (Figure S1 ; Hierarchical layout shows that F-types appear at terminal position in this Spoligoforest). 

### Clustering

A total of 98 clusters comprising 873 isolates (83.2%) were detected by using spoligotyping alone. By combining spoligotyping and MIRU profiles, 791 distinct combinations were obtained. The 98 combined clusters contained 355 isolates (33.9% of all isolates). The largest cluster, consisting of 20 isolates, belonged to SIT42/LAM9 lineage. All were Finnish patients. Only 10 of the 98 (10.2%) clusters contained both Finnish and foreign born patients. MIRU has a higher discriminatory power than spoligotyping, usually fragmenting spoligotype clusters. However, our results indicate that 39 MIRU patterns consisting of 220 isolates (5.4% of all MIRU patterns, 21.0% of all isolates) were divided by spoligotyping. Overall, 65 (25.0%) of the foreign born patients and 273 (37.2%) of the Finns were clustered by using spoligotyping and MIRU combined.

### Beijing lineage and drug resistance

Among the 1048 isolates analyzed, 117 (11.2%) were drug resistant and the most common resistance was to isoniazid only (28 out of 117 isolates/ 23.9%). Seventeen (1.6%) were multidrug resistant (MDR-TB). Out of the 17 MDR-TB cases, 7 cases (41.2%) were Finnish born of which one was ≥65 years old. Six out of the 17 isolates (35.3%) were clustered, and only one cluster contained more than one MDR-TB patient. 

## Discussion

This study provides an update of the molecular epidemiology of TB in Finland. The 4-year nationwide data showed that the majority of the TB cases were Finnish born and a large proportion was aged 65 or older. This is uncommon for a low TB incidence country, as can be seen, for example in Sweden or Denmark where the vast majority of TB cases are immigrants [2,4]. This is primarily explained by a different immigration situation of these countries, as in Sweden and Denmark 10% of the population is born abroad, while the corresponding figure in Finland is less than 3%. This could also be the reason why there seems to be less transmission in Finland between foreign and Finnish born cases, compared to Sweden and Denmark [3,4].

In Finland, a relatively high clustering rate (33.9%) was noted compared to Sweden (23.6% using MIRU-VNTR and spoligotyping) [12]. Clustering rate was higher among Finnish born than immigrant cases, which was also found in Denmark using a different methodology [4]. The interpretation of clustering in population based studies is complicated, particularly if it covers stable urban and rural populations. While clustering is associated with recent transmission in urban settings, it is more complicated to interpret on population levels due to possible predominance of certain well-preserved strains in the population [13,14]. Additionally, Finland – and other Nordic countries- had endured a very high tuberculosis incidence before the second world war [1]. As reported from Sweden and Norway, certain lineages (Haarlem and T) were predominately found among elderly cases, suggesting that these lineages were most likely associated with the epidemic during the first half of the 20^th^ century [15,16]. As found in this study, Haarlem and T were also the most common lineages found among Finnish born TB cases. The relatively high rate of clustering could partly be explained by well conserved strains in the Finnish population. 

The overall analysis shows that the isolates belonged to many different genotypic lineages, reflecting the wide variety of *M. tuberculosis* strains circulating in Finland. The most common lineages were T and Haarlem as in the case in the rest of Western Europe [10], although for many isolates the lineage was unknown. Based on phylogeographical analysis, the distribution of TB lineages in Finland seems to be similar to Sweden, but not to the other neighboring countries. The main difference between the lineages in Sweden and Finland was the presence of the Ural lineage in Finland (5.7% of isolates). The relatively recently named Ural lineage [17] was primarily found among Finnish born cases. This lineage is common in Estonia, Russia, and Latvia (Figure 2). Certain lineages such as Beijing, which is dominant in Russia, was barely seen in Finland 12 years ago and is now one of the more common lineages [9]. MDR-TB is still rare and most of the isolates belonged to the Beijing lineage. 

Almost all lineages were seen in both foreign born and Finnish born patients, but only one large cluster was shared by the two groups, suggesting that transmission of TB between Finns and foreigners does not occur frequently. In-depth cluster analysis and epidemiological research is warranted to investigate this conclusively. 

Although the majority of our isolates could be genotyped and classified successfully, many of the spoligotypes and MIRU profiles found in this study were not recognized in the international databases. We identified 68 isolates with spoligopatterns that were exclusively encountered in Finland (F-type), and of those 51 were orphan spoligotypes. This could be due to information lacking in the databases or could reflect the unique nature of Finnish *M. tuberculosis* isolates. These strains were genetically distinct from the more common SIT strains, but more research is needed to explore this in-depth. 

In conclusion, a large proportion of the *M. tuberculosis* isolates seen in Finland originated from Finnish born elderly patients. Moreover, many previously unidentified spoligotype profiles and isolates belonging to unknown lineages were encountered. Based on these study results, the TB situation in Finland is different from other low incidence countries. More research and in-depth assessment is needed to obtain a better insight in the unusual TB situation in Finland.

## Supporting Information

Figure S1 **Hierarchical layout of spoligotypes, Finland, 2008-2011.** A representation of parent to descendant spoligotypes within our study sample (n=1048 isolates) as seen through Spoligoforest trees drawn using the SpolTools software (available through http://www.emi.unsw.edu.au/spolTools), and reshaped and colored using the GraphViz software (available through: http://www.graphviz.org). The tree shown was drawn using a Hierarchical Layout where the F-types and orphan strains are highlighted in green. In this tree, each spoligotype pattern from the study is represented by a node with area size being proportional to the total number of isolates with that specific pattern. Changes (loss of spacers) are represented by directed edges between nodes, with the arrowheads pointing to descendant spoligotypes. The heuristic used selects a single inbound edge with a maximum weight using a Zipf model. Solid black lines link patterns that are very similar, i.e., loss of one spacer only (maximum weigh being 1.0), while dashed lines represent links of weight comprised between 0.5 and 1, and dotted lines a weight less than 0.5. Note that SIT53/T1 constitutes the biggest node (n=135), followed by SIT47/H1 (n=65), SIT1/Beijing (n=55), SIT49/H3 (n=43) and SIT50/H3 (n=40), which are other predominant patterns in Finland. On the other hand, F-types and/or orphan isolates appear mostly at terminal positions on the tree, or as isolated strains without interconnections with the other strains.
(PDF)

Table S1 **Orphan and F-type strains (n=68) and corresponding spoligotyping.**
(XLSX)

Table S2 **Overview of spoligotypes found in Finland, 2008-2011.**
(XLSX)
