# Supplementary figures and images for: Molecular Epidemiology of Tuberculosis in Finland, 2008-2011
Source: PLoS One. 2013 Dec 26;8(12):e85027. doi: 10.1371/journal.pone.0085027 (PMC3873426; doi:10.1371/journal.pone.0085027)

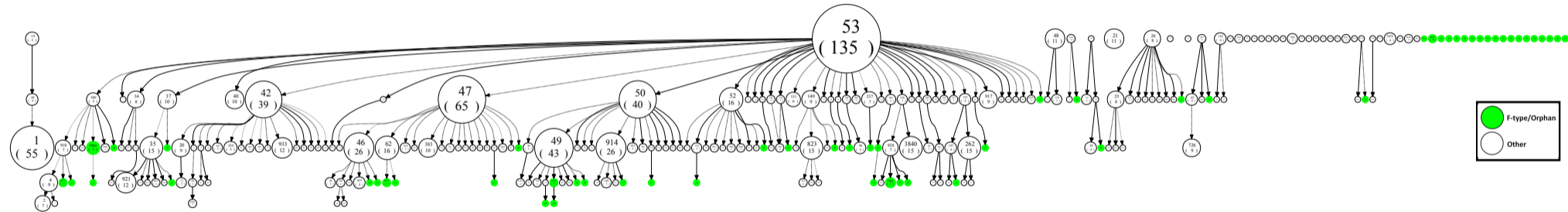

Supplement: Figure S1 — Hierarchical layout of spoligotypes, Finland, 2008-2011. A representation of parent to descendant spoligotypes within our study sample (n=1048 isolates) as seen through Spoligoforest trees drawn using the SpolTools software (available through http://www.emi.unsw.edu.au/spolTools), and reshaped and colored using the GraphViz software (available through: http://www.graphviz.org). The tree shown was drawn using a Hierarchical Layout where the F-types and orphan strains are highlighted in green. In this tree, each spoligotype pattern from the study is represented by a node with area size being proportional to the total number of isolates with that specific pattern. Changes (loss of spacers) are represented by directed edges between nodes, with the arrowheads pointing to descendant spoligotypes. The heuristic used selects a single inbound edge with a maximum weight using a Zipf model. Solid black lines link patterns that are very similar, i.e., loss of one spacer only (maximum weigh being 1.0), while dashed lines represent links of weight comprised between 0.5 and 1, and dotted lines a weight less than 0.5. Note that SIT53/T1 constitutes the biggest node (n=135), followed by SIT47/H1 (n=65), SIT1/Beijing (n=55), SIT49/H3 (n=43) and SIT50/H3 (n=40), which are other predominant patterns in Finland. On the other hand, F-types and/or orphan isolates appear mostly at terminal positions on the tree, or as isolated strains without interconnections with the other strains. (PDF) [file pone.0085027.s001.pdf]
